# Sarcoïdose cutanée sur cicatrice: forme habituellement évolutive (à propos d’un cas)

**DOI:** 10.11604/pamj.2021.39.268.27864

**Published:** 2021-08-24

**Authors:** Madiha El Jazouly, Fatimzahra Chahboun, Awatef Kelati, Mounia El Omari, Abderahman Al Bouzidi, Soumiya Chiheb

**Affiliations:** 1 Service de Dermatologie, Hôpital Cheikh Khalifa Ibn Zaid, Université Mohammed VI des Sciences de la Santé de Casablanca, Casablanca, Maroc,; 2 Service de Chirurgie Plastique, Hôpital Cheikh Khalifa Ibn Zaid, Université Mohammed VI des Sciences de la Santé de Casablanca, Casablanca, Maroc,; 3 Service d´Anatomopathologie, Hôpital Cheikh Khalifa Ibn Zaid, Université Mohammed VI des Sciences de la Santé de Casablanca, Casablanca, Maroc

**Keywords:** Sarcoïdose, cicatrice, sarcoïdose pulmonaire, à propos d’un cas, Sarcoidosis, scar, pulmonary sarcoidosis, case report

## Abstract

La sarcoïdose est une affection granulomateuse systémique dont les manifestations cutanées sont fréquentes pouvant être une circonstance de découverte très originale d´une sarcoïdose évolutive sur le plan viscéral. Nous rapportons un cas de sarcoïdose pulmonaire révélée par la réactivation d´une cicatrice cutanée suite à un traumatisme survenu 20 ans plus tôt. Le bilan radiologique a mis en évidence un aspect en faveur d´une sarcoïdose médiastino-pulmonaire stade 2. Le diagnostic de sarcoïdose doit être envisagé devant toute modification récente d´une cicatrice afin d´établir une prise en charge précoce.

## Abstract (en)

The sarcoidosis is a systemic granulomatous disease. It is usually characterized by skin manifestations which may be suggestive of progressive sarcoidosis with visceral involvement. We here report a case of pulmonary sarcoidosis revealed by the reactivation of an old cutaneous scar following a trauma occurred 20 years earlier. Radiological assessment showed mediastino-pulmonary sarcoidosis stage 2. The diagnosis of sarcoidosis should be suspected in patients with any recent scar modification in order to establish early management.

## Introduction

La sarcoïdose ou maladie de Besnier-Boek Schumann est une affection granulomateuse systémique d´étiologie inconnue. Une réponse immunitaire exagérée à différents stimuli antigéniques pourrait jouer un rôle dans la formation d´un granulome épithelio-gigantocellulaire sans nécrose caséeuse, caractéristique de cette affection [1]. La sarcoïdose touche principalement les poumons, les ganglions lymphatiques (dans 80 à 90% des cas), l´os, le foie, la rate, les glandes parotides, les yeux et la peau. Les localisations cutanées moins fréquentes, sont observées selon les séries entre 25 et 35% des cas [1, 2]. La réactivation des cicatrices préexistantes reste une présentation relativement rare de la maladie. Elles peuvent être la première conséquence du processus inflammatoire révélant ainsi une pathologie systémique potentiellement évolutive. Nous rapportons un cas de sarcoïdose cutanée sur cicatrice révélant une atteinte pulmonaire.

## Patient et observation

**Présentation du patient**: Mme C.S, âgée de 57 ans, Diabétique type 2 sous insuline, était victime d´un accident de la voie publique à l´âge de 34 ans avec une plaie ouverte au niveau du front, la main gauche et du cou avec une cicatrisation complète sans chéloïdes ni cicatrice hypertrophique. L´évolution était marquée par l´apparition depuis 9 mois (20 ans après) d´une hypertrophie inflammatoire des lésions cicatricielles; jusque-là indemnes; indolores non prurigineuses motivant ainsi la consultation.

**Figure 1:**
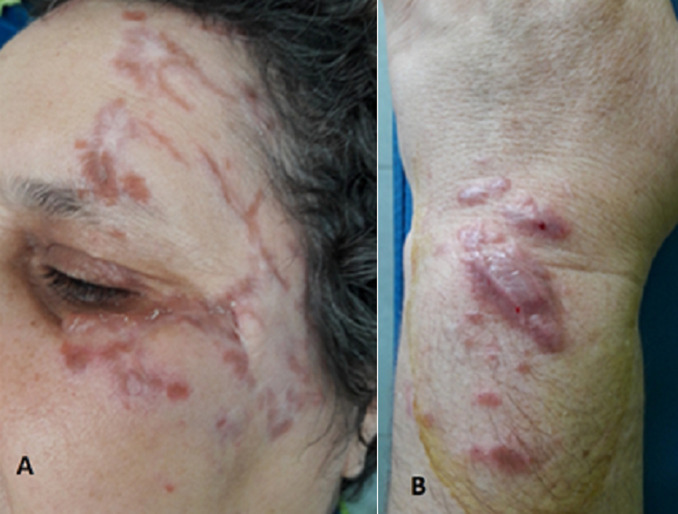
infiltration en plaques papulonodulaires érythémateuses, pourpres des anciennes cicatrices de la face (A) et du poignet droit (B)

**Résultats cliniques**: l´examen clinique montrait des lésions papulonodulaires érythématoviolacées infiltrées et indolores de deux cicatrices (l´une au niveau fronto-temporale gauche et l´autre au niveau du dos de la main gauche) avec apparition de nouvelles papules érythémateuses au niveau du dos de la main droite (Figure 1). Le reste de l´examen physique était sans particularités.

**Figure 2:**
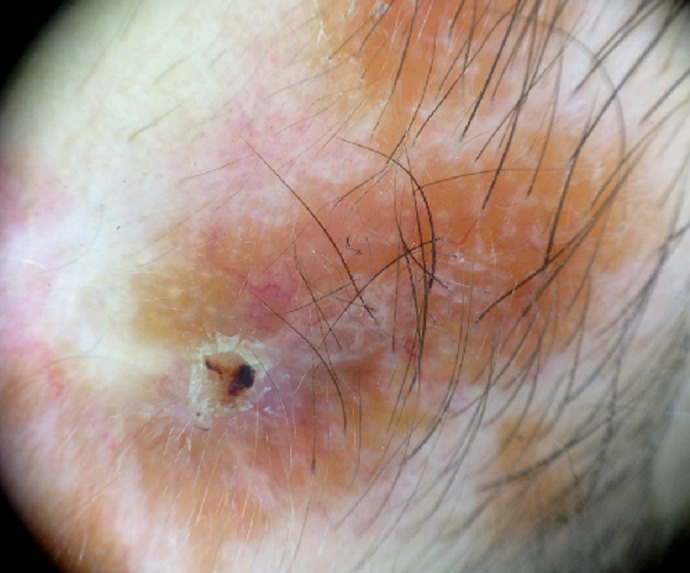
dermoscopie: vaisseaux linéaires arborisants avec des zones jaunes orangées

**Figure 3:**
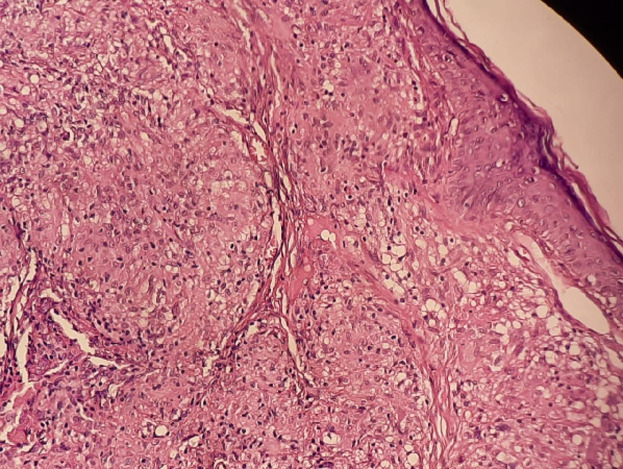
biopsie cutanée: granulomes épithélioïdes et gigantocellulaires sans nécrose caséeuse HES (coloration hématoxyline et éosine)

**Figure 4:**
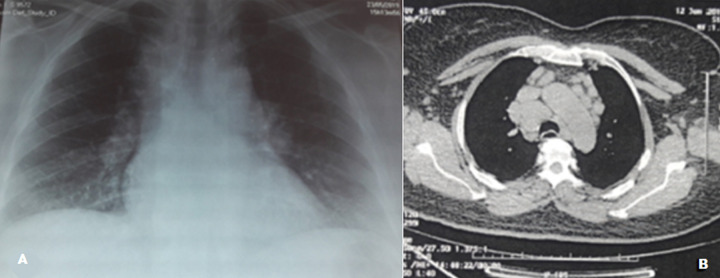
sarcoïdose médiastinopulmonaire stade II; A) radio du thorax avec élargissement médiastinal et un infiltrat interstitiel bilatéral; b) scanner thoracique montrant des adénopathies hilaires bilatérales avec atteinte parenchymateuse

**Démarche diagnostique**: la dermoscopie montrait des zones orangées avec des vaisseaux linéaires arborisants (Figure 2). La biopsie cutanée révélait une dermite granulomateuse folliculaire épithélio- gigantocellulaire cadrant avec une sarcoïdose cutanée (Figure 3). Le bilan biologique notait une hypercalcémie (110; valeur normale: 85-101 milligramme par litre) avec élévation de l´enzyme de conversion de l´angiotensine (156; valeur normale : 20-70 unité par litre). La radio de thorax complétée par une tomodensitométrie thoracique mettait en évidence un aspect en faveur d´une sarcoïdose médiastino-pulmonaire stade 2 (Figure 4A, Figure 4B). Le diagnostic de sarcoïdose cutanée et pulmonaire était retenu devant des arguments cliniques, biologiques, histologiques et radiologiques.

**Intervention thérapeutique**: un traitement à base d´antipaludéens de synthèse (APS): Plaquenil à raison de 2 comprimés par jour, et des injections intra lésionnelles de corticoïde étaient instaurés.

**Suivi et résultats des interventions**: on a noté une évolution favorable avec affaissement des lésions après 3 mois de traitement. Une surveillance clinique, biologique et radiologique a été instaurée. Aucune nouvelle manifestation n´a été notée avec un recul 2 ans.

**Consentement éclairé**: la patiente a donné son consentement éclairé.

## Discussion

La survenue d´une sarcoïdose sur cicatrice cutanée est une entité connue depuis la fin du 19^e^ siècle. Cependant, elle reste rare. Sa fréquence est estimée à 5% et touche avec prédilection les femmes d´âge moyen [1, 3]. Plusieurs types de cicatrices peuvent être le site de cette maladie granulomateuse: des points d´une intervention chirurgicale (blépharoplastie, hystérectomie…), une brulure, un site d´injection intraveineuse ou d´intradermoréaction, une cicatrice d´herpès ou de zona ou encore une cicatrice ancienne d´un traumatisme comme c´est le cas de notre patiente. Le délai entre le traumatisme et la modification de la cicatrice est en moyenne de 20 ans avec des extrêmes allant de quelques semaines à quelques dizaines d´années [3, 4]. La sarcoïdose est une maladie systémique caractérisée sur le plan cutané par son polymorphisme lésionnel. Il peut s´agir de manifestations non spécifiques réactionnelles dominées par l´érythème noueux, ou des lésions spécifiques d´expressions cliniques très variées .caractérisées par la présence des grains lupoïdes à la vitro-pression. Des formes rares souvent trompeuses ont également été décrites comme les formes psoriasiformes, ichtyosiformes ou érythrodermiques d´où son appellation « la grande simulatrice ».

L´aspect clinique des lésions cicatricielles est souvent évocateur, consistant en l´apparition soudaine de nodules et d´infiltrations indolores et non prurigineuses. Il peut s´agir de lésions pseudo- chéloïdiennes infiltrées, saillantes rouges ou violacées, de plaques angiolupoïdes, de nodules dermiques fermes, élastiques, de type sarcoïdes à petits nodules ou de nodules d´hypodermite plus profonde [5]. L´existence d´autres lésions cutanées à distance est évocatrice du diagnostic. Néanmoins, cette situation n´est pas obligatoire pour retenir le diagnostic. La dermoscopie a prouvé son utilité dans la sarcoïdose. En effet, elle permet une meilleure visualisation des structures dermiques vasculaires et éventuellement la présence des granulomes en mettant en évidence des globules jaune-orangés translucides ou des zones sans structure. L´étiopathogénie de la sarcoïdose ainsi que la réactivation du tissu cicatriciel est mal comprise, suggérant plusieurs hypothèses. La formation du granulome serait le résultat d´une réaction d´hypersensibilité à des corps étrangers survenant chez des patients ayant une prédisposition génétique [5, 6]. La sarcoïdose cutanée est fréquemment associée à une atteinte médiastinale. Bien que son apparition sur une cicatrice témoigne habituellement d´une maladie évolutive sur le plan viscéral, elle peut également être inaugurale de la maladie et précéder l'atteinte pulmonaire dans 30% des cas [7, 8], ou encore être concomitante d´une de ses poussées. Le délai d´apparition d´éventuelles manifestations systémiques varie d´un mois à un an. Ainsi, il est recommandé de réaliser de manière périodique un examen clinique, un bilan biologique, radiologique avec électrocardiogramme et examen ophtalmologique. Notre patiente a présenté une infiltration de multiples cicatrices cutanées d´un traumatisme très ancien de plus de 20 ans, révélant une atteinte pulmonaire stade II qui associe aux adénopathies médiastinales, une atteinte parenchymateuse à type de pneumopathie interstitielle.

Lorsque l´atteinte systémique existe, il n´y a aucune corrélation entre l´étendue des lésions cutanées et la sévérité de la maladie. En outre, certains auteurs ont précisé que le risque varie selon le type lésionnel. Il est plus important dans le lupus pernio, les formes en plaques, à gros nodules, hypodermiques, ainsi que dans les atteintes du cuir chevelu et des paupières. En revanche, le risque paraît faible dans les formes à petits nodules. Deux scores d´évaluation de l´activité et de la sévérité de l´atteinte cutanée sont désormais disponibles et vont permettre d´évaluer l´effet des nombreux traitements disponibles avec objectivité [9]. Le traitement de l´atteinte cutanée au cours de la sarcoïdose reste codifié reposant essentiellement sur la corticothérapie locale, intra-lésionnelle et par voie générale associée en fonction des formes cliniques, aux antipaludéens de synthèses et au méthotrexate. Toutefois ces traitements sont parfois inefficaces, notamment, dans certaines formes récalcitrantes, ce qui explique le recours à d´autres moyens thérapeutiques (le thalidomide, la biothérapie comme l´aprémilast ou encore les anti-TNF alpha) [10]. En revanche, la sarcoïdose cutanée sur cicatrice régresse souvent spontanément et l´injection de corticostéroïdes en intralesionnel ou le tacrolimus topique représentent une bonne alternative thérapeutique.

## Conclusion

Malgré que la sarcoïdose est loin d´être l´unique dermatose ayant un tropisme électif pour les cicatrices cutanées, ce diagnostic doit être envisagé devant toute modification récente d´une cicatrice ainsi qu´une surveillance périodique afin de détecter précocement les manifestations systémiques.
